# Atrial fibrillation burden: a new outcome predictor and therapeutic target

**DOI:** 10.1093/eurheartj/ehae373

**Published:** 2024-07-02

**Authors:** Nina Becher, Andreas Metzner, Tobias Toennis, Paulus Kirchhof, Renate B Schnabel

**Affiliations:** Department of Cardiology, University Heart & Vascular Center Hamburg, University Medical Center Hamburg-Eppendorf, Martinistrasse 52, 20246 Hamburg, Germany; German Center for Cardiovascular Research (DZHK), Partner Site Hamburg/Kiel/Luebeck, Postdamer Str. 58, 10785 Berlin, Germany; Department of Cardiology, University Heart & Vascular Center Hamburg, University Medical Center Hamburg-Eppendorf, Martinistrasse 52, 20246 Hamburg, Germany; German Center for Cardiovascular Research (DZHK), Partner Site Hamburg/Kiel/Luebeck, Postdamer Str. 58, 10785 Berlin, Germany; Department of Cardiology, University Heart & Vascular Center Hamburg, University Medical Center Hamburg-Eppendorf, Martinistrasse 52, 20246 Hamburg, Germany; German Center for Cardiovascular Research (DZHK), Partner Site Hamburg/Kiel/Luebeck, Postdamer Str. 58, 10785 Berlin, Germany; Department of Cardiology, University Heart & Vascular Center Hamburg, University Medical Center Hamburg-Eppendorf, Martinistrasse 52, 20246 Hamburg, Germany; German Center for Cardiovascular Research (DZHK), Partner Site Hamburg/Kiel/Luebeck, Postdamer Str. 58, 10785 Berlin, Germany; Institute of Cardiovascular Sciences, University of Birmingham, Birmingham, UK; Department of Cardiology, University Heart & Vascular Center Hamburg, University Medical Center Hamburg-Eppendorf, Martinistrasse 52, 20246 Hamburg, Germany; German Center for Cardiovascular Research (DZHK), Partner Site Hamburg/Kiel/Luebeck, Postdamer Str. 58, 10785 Berlin, Germany

**Keywords:** Atrial fibrillation, Burden, Oral anticoagulation, Stroke, Rhythm control

## Abstract

Atrial fibrillation (AF), the most common sustained cardiac arrhythmia, is not a dichotomous disease trait. Technological innovations enable long-term rhythm monitoring in many patients and can estimate AF burden. These technologies are already used to detect and monitor AF. This review describes the relation between AF burden and outcomes and potential effects of AF burden reduction. A lower AF burden is associated with a lower risk of stroke and heart failure in patients with AF: stroke risk without anticoagulation is lower in patients with device-detected AF and a low AF burden (stroke rate 1%/year) than in patients with persistent and permanent AF (stroke rate 3%/year). Paroxysmal AF shows intermediate stroke rates (2%/year). Atrial fibrillation burden–reducing interventions can reduce cardiovascular outcomes in patients with AF: early rhythm control reduces cardiovascular events including stroke and heart failure in patients with recently diagnosed AF and cardiovascular conditions. In patients with heart failure and AF, early rhythm control and AF ablation, interventions that reduce AF burden, reduce mortality and heart failure events. Recent technological innovations allow to estimate AF burden in clinical care, creating opportunities and challenges. While evidence remains limited, the existing data already suggest that AF burden reduction could be a therapeutic goal. In addition to anticoagulation and treatment of cardiovascular conditions, AF burden reduction emerges as a therapeutic goal. Future research will define the AF burden that constitutes a relevant risk of stroke and heart failure. Technologies quantifying AF burden need careful validation to advance the field.

## Graphical Abstract

**Graphical Abstract:**
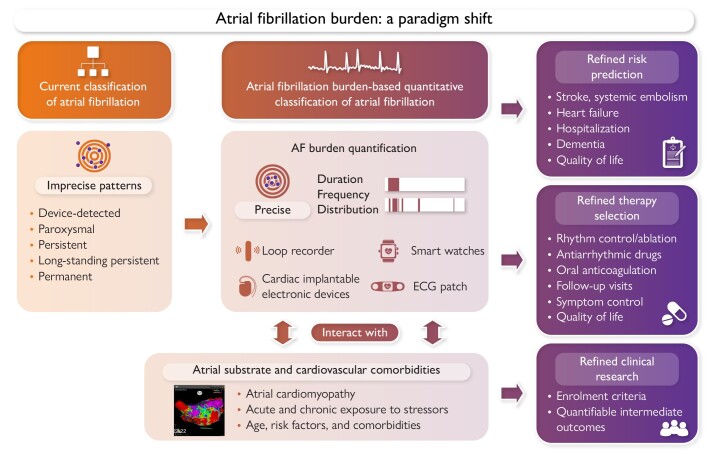
Clinical classification of atrial fibrillation (AF) and quantitative classification of AF using AF burden and its interaction and influence on outcomes, precision therapy, and clinical trials and research.

## Introduction

Atrial fibrillation (AF) is a common cause of stroke, heart failure, cardiovascular death, myocardial infarction, and dementia.^1–4^ In addition, AF decreases quality of life through symptoms^4,5^ and psychological distress.^6^ A rising prevalence of AF will further increase the burden of AF and AF-related complications on healthcare.^7^ Oral anticoagulation^8^ and, more recently, early rhythm control therapy^9,10^ improve outcomes in patients with AF by preventing stroke, cardiovascular death, heart failure hospitalizations, and acute coronary syndrome. Epidemiological, observational, and interventional research typically assume that the risk of these events depends on comorbidities without differences based on AF pattern. Recent trial outcomes challenge this simple concept: the outcome-reducing effect of early rhythm control therapy, linked to attaining sinus rhythm,^11^ supports the concept that less time in AF can reduce outcomes. The rate of stroke is low (1.1%–1.2%/year) without anticoagulation in patients with very rare and short episodes of device-detected AF (DDAF) despite multiple clinical stroke risk factors, and the effect of anticoagulation is weak.^12–14^ At the same time, the growing availability of consumer electronics equipped with increasingly reliable algorithms to monitor cardiac rhythm, including smartphones and wearables,^15–17^ offers accessible methods to detect rare and short episodes of AF and to estimate arrhythmia burden.^18^ This review summarizes the recent data on AF burden and its relation to outcomes, outlines findings that can help today’s shared decision-making with patients with AF, and identifies research and innovation opportunities.

### Natural progression and regression of atrial fibrillation

**Figure 1:**
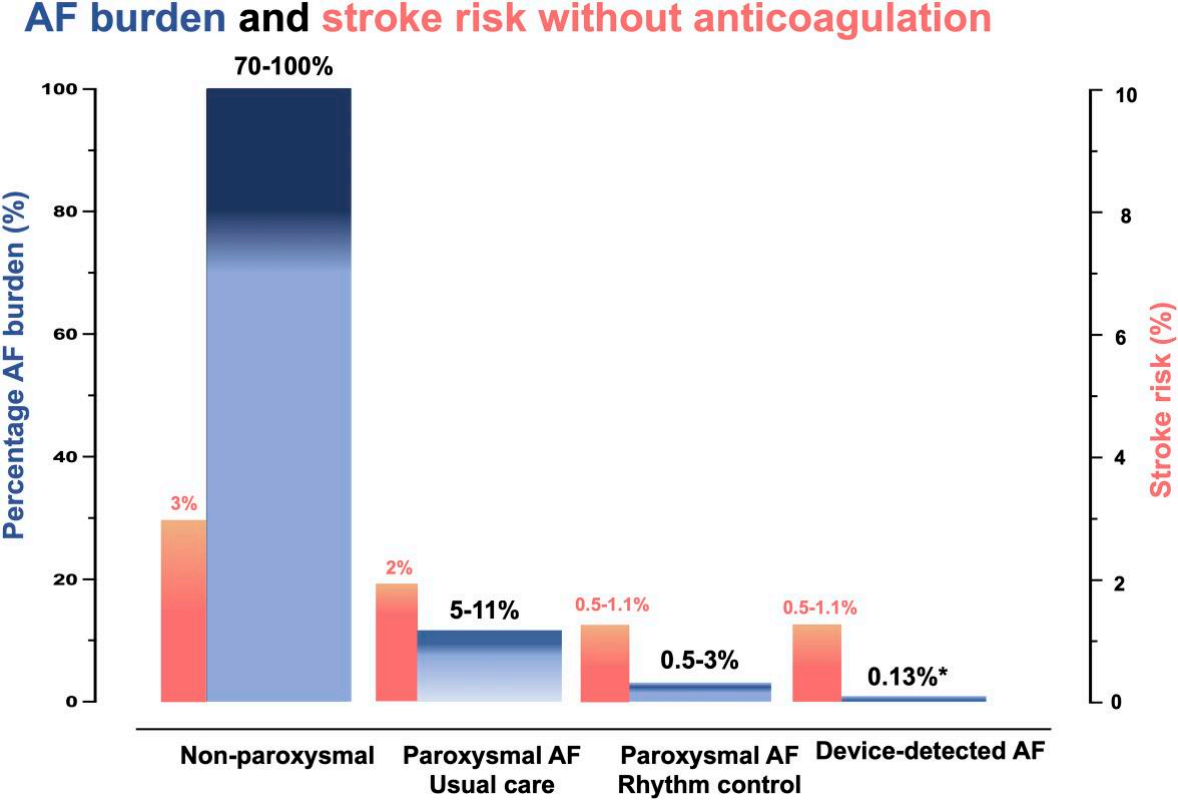
The estimated risk of stroke associated with atrial fibrillation burden or atrial fibrillation pattern. *According to the data of the LOOP study^29^ with median atrial fibrillation burden of 0.13% (interquartile range 0.03–1.05). Atrial fibrillation burden was defined as cumulative duration of all atrial fibrillation episodes lasting >6 min from the first adjudicated atrial fibrillation episode onward, divided by total duration of monitoring^29^

The initial presentation of patients with newly diagnosed AF can be paroxysmal, persistent, or with an AF pattern that still requires determination (‘first diagnosed’). In the EAST-AFNET 4 (Early Treatment of Atrial Fibrillation for Stroke Prevention Trial) trial, an outcome trial that enrolled only patients with recently diagnosed AF, approximately one-third of the patients presented with each of these three AF patterns.^9^ When AF is established, typically when rhythm control treatment failed, AF is classified as permanent.^1^ Atrial fibrillation often progresses from paroxysmal, self-terminating episodes to persistent AF. On average, this progression is slow: ∼1 in 20 patients (5%/year) will experience progression from paroxysmal to persistent AF per year,^19^ and more than half of patients with paroxysmal AF without concomitant conditions do not progress over a 25 year period.^20^ Recent clinical research using continuous rhythm monitoring shows that many patients with AF do not show any recurrences in 1 year of follow-up and some even regress, showing paroxysmal AF after periods of persistent AF.^21,22^ This regression of AF patterns is not common, but consistently found.^23^ The average AF burden in patients with paroxysmal AF is ∼11% when monitored using implanted devices,^24^ and similar estimates are found when days in AF are counted in patients with paroxysmal AF undergoing daily telemetric electrocardiogram (ECG) monitoring.^25^ The daily AF burden in RACE-V increased from 3.2% to 3.8%, 5.2%, and 6.1% in patients with paroxysmal AF undergoing continuous rhythm monitoring by implanted devices.^22^ In RACE-V, AF progressed to non-paroxysmal patterns in some patients (5.5%/year), some patients regressed, and the overall AF burden remained low.^26^ Variable recurrences of AF and the slow and varying progression of AF can explain that 60% of patients randomized to usual care in the EAST-AFNET 4 trial were in sinus rhythm at 2 years^9,27^ and that AF ablation retains its efficacy after a 12-month waiting period.^28^ Conceptually, the AF burden should be 100% in patients with non-paroxysmal AF, but spontaneous regression reduces their arrhythmia burden to 70%–100%^24^ (*Figure 1*).

#### Device-detected atrial fibrillation

Systematic Holter ECG recordings first showed that short atrial arrhythmias, including atrial runs and frequent atrial ectopy, can be precursors of AF.^30,31^ Such short atrial arrhythmias are associated with a risk of AF, stroke, and death.^31^ Different terms were used to describe short and rare atrial arrhythmias, including micro-AF, subclinical AF, and atrial high-rate episodes. In the NOAH-AFNET 6 (Non-Vitamin K Antagonist Oral Anticoagulants in Patients with Atrial High Rate Episodes) trial, almost all (97%) atrial high-rate episodes documented at enrolment were confirmed to be AF by an experienced core lab.^32^ The analysis of DDAF episodes helped to improve our understanding of the development of AF: DDAF is found 10 times more (∼30% of patients with implanted devices and stroke risk factors)^33–35^ than ECG-diagnosed AF. Many patients only have a few short episodes of DDAF. In the LOOP (Atrial Fibrillation Detected by Continuous ECG Monitoring Using Implantable Loop Recorder to Prevent Stroke in High-risk Individuals) study, the median AF burden was 0.13%, 16% of patients in LOOP progressed to episodes lasting longer than 24 h, but 22% did not have repeat episodes in the last 6 months or longer of monitoring.^29^ Two large randomized trials with 6-monthly ECGs during clinic visits consistently show a slow progression from DDAF to ECG-diagnosed AF [ARTESiA (Apixaban for the Reduction of Thrombo-Embolism in Patients with Device-Detected Subclinical Atrial Fibrillation), 6%/year including advancement to long episodes of DDAF^36^; NOAH-AFNET 6, 9%/year^13^]. The slightly higher rate of progression in NOAH-AFNET 6 may be due to the shorter follow-up time and inclusion of patients with longer episodes in NOAH-AFNET 6.^13,36^

### The interplay of atrial fibrillation burden, comorbidities, and outcomes

Atrial fibrillation and comorbidities interact in complex ways,^37^ modulating the rate of AF progression and the risk of AF-related outcomes. Comorbidities accelerate the progression of AF. Lack of comorbidities appears to reduce the rate of AF progression by two-thirds from 5%/year in average populations with AF^19^ to 1%–2%/year.^20^ Furthermore, the burden of DDAF appears higher in patients with more comorbidities, estimated by a higher CHADS_2_ (heart failure, hypertension, age ≥ 75 years, diabetes mellitus, stroke, or transient ischaemic attack) score.^38^ A large observational study linking device data with outcomes in electronic health records suggests that a higher burden of DDAF and a very high CHA_2_DS_2_-VASc score (heart failure, hypertension, age ≥ 75 years, diabetes mellitus, prior stroke or transient ischaemic attack, vascular disease, age 65–74 years, and female) could be associated with a higher risk of stroke and systemic embolism.^39^ The effect of comorbidities on stroke risk was weak in the NOAH-AFNET 6 trial,^40^ and very long DDAF episodes ≥24 h were not associated with a higher CHA_2_DS_2_-VASc score in that data set.^13^ Thus, the interaction between DDAF and comorbidities is subtle and probably not linear.^41^

At the same time, presence of AF also enhances the risk of heart failure events, stroke, and cardiovascular death (*Table 1*).^1^

1. *Atrial fibrillation and left ventricular function*. Atrial fibrillation directly impairs ventricular function^49^ and can even result in arrhythmia-induced cardiomyopathy^50^ that will reverse after restoration of normal sinus rhythm using AF ablation.^51–53^ In other patients, heart failure can predispose to AF,^54,55^ and AF and heart failure share genetic and acquired pathomechanisms.^56^ A high AF burden was associated with more heart failure events in a large population of patients with paroxysmal AF.^57^ There is a strong bidirectional association between heart failure and AF burden, where heart failure can contribute to AF, and vice versa.^58^ Controlled clinical trials of rhythm control and AF ablation, interventions that lower AF burden, reduce heart failure events in patients with reduced left ventricular function and AF.^51,59,60^ Ongoing studies testing AF ablation will determine whether reducing AF burden can improve outcomes in patients with heart failure with preserved ejection fraction (HFpEF) and AF (NCT055008256).
2. *Atrial fibrillation and stroke*. Reduced flow in the left atrium and especially in the left atrial appendage, endothelial dysfunction, and activation of local and systemic prothrombotic signalling combine to enhance thrombogenesis in the left atrium and cardioembolic stroke in patients with AF.^37,61,62^ This leads to cardioembolic strokes that are commonly found in patients with AF.^1^ The incidence of cardioembolic strokes can be significantly reduced by oral anticoagulants.^8,63^ Left atrial appendage occlusion^64^ and early rhythm control therapy^9^ each reduce stroke by one-third on top of anticoagulation, demonstrating a prominent atrial contribution to stroke. On the other hand, atrial arrhythmia may be more frequent in the acute phase after a stroke.^65^ Thus, interactions between comorbidities and AF appear to create an atrial prothrombotic milieu.
3. *Cardiovascular death* can be due to AF and occur independent of AF.^66^ The reductions in mortality seen in anticoagulation trials^63,67^ suggest that stroke prevention can reduce cardiovascular deaths. The effects of early rhythm control^9^ and AF ablation^51,60^ on cardiovascular death and total mortality suggest that rhythm control can have similar effects, probably by preventing stroke and heart failure events. Overall, these effects of AF on cardiovascular death compete with other diseases causing cardiovascular death, and the contribution of AF on cardiovascular death will depend on the number, intensity, and quality of treatment of other cardiovascular diseases.

**Table 1:** Overview of studies and randomized controlled trials in patients with device-detected atrial fibrillation and screening-detected atrial fibrillation and their relation of atrial fibrillation burden/duration of the longest device-detected atrial fibrillation episode on outcomes

Study/data set	AF type or pattern (paroxysmal, persistent, permanent) or device-detected AF (DDAF)		Age (years)	Women (%)	Comorbidity burden (CHA_2_DS_2_-VASc score)	Duration of longest DDAF episode^a^ or (%/monitored time)	Event rate of stroke^b^ (and SE; %/patient-year)	Event rate of HF (%/patient-year)	Event rate of CV death (%/patient-year)
Device-detected AF									
NOAH-AFNET 6 (Kirchhof *et al.*^12^)									
Anticoagulation (DOAC)	Device-detected AF (pacemakers, defibrillators, ILR)		77.4 ± 6.5	36.9	4 (3–5)	Median 2.8 h	0.9 (ischaemic stroke)	0.5 (death due to HF)	2.0
No anticoagulation (placebo)			77.5 ± 6.8	37.9	4 (3–5)		1.1 (ischaemic stroke)	0.6 (death due to HF)	2.2
ARTESiA (Healey *et al.*^14^)									
Anticoagulation (DOAC)	Device-detected AF(pacemakers, defibrillators, ILR)		76.9 ± 7.6	35.7	3.9 ± 1.1	Median 1.47 h	0.64 (ischaemic stroke)		1.47
No anticoagulation (aspirin)			76.7 ± 7.7	36.5	3.9 ± 1.1		1.02 (ischaemic stroke)		1.53
Carelink+OPTUM (Kaplan *et al.*^39^)									
Anticoagulation (DOAC)	Device-detected AF (pacemakers, defibrillators)		71.8 (10.4)	37	4.4 (1.8)	No AF	0.81 (stroke and SE)		
						6 min–23.5 h	1.0 (stroke and SE)		
						>23.5 h	1.43 (stroke and SE)		
No anticoagulation			68.6 (12.7)	35	3.0 (2.0)	No AF	0.33–1.79^c^ (stroke and SE)		
						6 min–23.5 h	0.53–2.21^c^ (stroke and SE)		
						>23.5 h	0.86–1.77^c^ (stroke and SE)		
Li *et al.*^42^	Device-detected AF		72 ± 13	39.4	3.4 ± 1.6		0.74 (ischaemic stroke); 1.85 (ischaemic stroke, TIA, SE)	5	
ACTIVE-A and AVERROES (Vanassche *et al.*^43^)									
Aspirin	Baseline ECG, rhythm strip, or minimum 30 min of device-detected AF	Paroxysmal	69.0 ± 9.9	47.7	3.1 ± 1.4		2.1 (stroke and SE)		
		Persistent	68.6 ± 10.2	42.3	3.1 ± 1.4		3.0 (stroke and SE)		
		Permanent	71.9 ± 9.8	39.8	3.6 ± 1.5		4.2 (stroke and SE)		
SOS (Boriani *et al.*^44^)	Device-detected AF (pacemakers, defibrillators)		70 (61, 76)	31	CHA_2_DS_2_-VASc score ≥2: 59%	≥5 min–1 h	0.08		
						1–6 h	0.34		
						≥6–<12 h	0.26		
						≥12–23 h	0.30		
						>23 h	0.23		
ASSERT (Van Gelder *et al.*^45^)	Device-detected (AF pacemakers, defibrillators)		77.7 ± 7	43.7	2.2 ± 1.1 (CHADS_2_)		1.52 (ischaemic stroke); 1.69 (stroke and SE)	3.07 (hospitalization for HF)	2.92
TRENDS (Glotzer *et al.*^46^)	Device-detected AF (pacemakers, defibrillators)		70.9 ± 11.1	33.6	2.2 ± 1.2	5.5 h (median 30-day window)	1.1 (stroke/TIA SE, low AT/AF burden <5.5 h); 2.4 (stroke/TIA/SE, high AT/AF burden 5.5 h)		
Screening-detected AF									
STROKESTOP (Svennberg *et al.*^47^)									
Invited to screening group^d^	Screening-detected AF (handheld single-lead ECG twice a day for 2 weeks or other means of long-term ECG)	75–76	53.9–55.4	3.3–3.7 (1.1–1.4)		0.9		1.39	
Standard care	Annual interview and standard contact general practitioner					0.98		1.38	
LOOP (Svendsen *et al.*,^48^ Diederichsen *et al.*^29^)									
	Standard care	74.7 ± 4.1	47.3	4 (3–4)		1.39 (stroke, SE, TIA; 13.1% oral anticoagulation initiated)			0.67
	Screening-detected AF (ILR)	74.7 ± 4.1	47.2	4 (3–4)	0.13% (DDAF burden^e^)	1.27 (stroke, SE, TIA; 29.2% oral anticoagulation initiated)			0.55

Age and CHA_2_DS_2_-VASc score are shown as mean ± standard deviation or median (interquartile range) or percentage of patients. Anticoagulation was often not initiated in older data sets and/or that atrial fibrillation was not systematically documented by ECG. Further information on event rates in other, smaller reports on patients with device-detected AF can be found in the design papers of NOAH-AFNET 6 and ARTESiA.

AF, atrial fibrillation; CV, cardiovascular; DDAF, device-detected atrial fibrillation, DOAC, direct oral anticoagulant; ECG; electrocardiogram; h, hours; HF, heart failure; ILR, implantable loop recorder; IQR, interquartile range; SE, systemic embolism; SD, standard deviation; TIA, transient ischaemic attack.

^a^The duration of the longest device-detected AF episode can be viewed as a proxy for AF burden.

^b^Stroke is differently defined.

^c^Different risk according to CHA_2_DS_2_-VASc score.

^d^Approximately half of the participants invited for active AF screening did not participate, diluting the effect of ECG screening.

^e^Derived from Diederichsen *et al.*^29^ and defined as cumulative duration of all AF episodes lasting 6 min from the first adjudicated AF episode onward, divided by total duration of monitoring.

Atrial fibrillation also affects symptoms, quality of life, and other outcomes.^1^ Its effects on dementia^3,68^ and quality of life^5,69–73^ are covered elsewhere and will not be discussed in detail in this paper.

### Rhythm control therapy reduces atrial fibrillation burden and prevents atrial fibrillation–related outcomes

Rhythm control therapy using antiarrhythmic drugs and, more effectively, AF ablation prevents AF recurrences, prolongs the time to recurrent AF, and reduces the AF burden.^71,74,75^ Atrial fibrillation ablation lowers the average AF burden to <1%, while antiarrhythmic drug therapy appears to reduce AF burden to 1%–3%^73,76–78^ (*Figure 1*). Rhythm control also slows the progression from paroxysmal to persistent AF.^79–81^ Atrial fibrillation ablation slows AF progression more effectively than antiarrhythmic drug therapy.^5,78,81^ Intensive treatment of concomitant conditions has a smaller effect on recurrent AF^82^ and outcomes.^83^ The concept that reducing AF burden can reduce cardiovascular events^41^ has been revived by the outcome-reducing effect of systematic early rhythm control therapy in the EAST-AFNET 4 trial^9^ and by smaller AF ablation trials in patients with heart failure.^59,60^ The safety and the effectiveness of early rhythm control have been replicated in several non-randomized analyses of large healthcare data sets.^84–86^ They are aligned with the outcome-improving effect of AF ablation in patients with heart failure and reduced ejection fraction^59^ and in patients on a waiting list for heart transplantation.^60^ Similarly, outcome-reducing effects of rhythm control therapy with dronedarone were already observed in the ATHENA (A Placebo-Controlled, Double-Blind, Parallel Arm Trial to Assess the Efficacy of Dronedarone 400 mg bid for the Prevention of Cardiovascular Hospitalization or Death from Any Cause in Patients with Atrial Fibrillation/Atrial Flutter) trial,^87^ including a reduction of stroke.^88^ The neutral results of the older AFFIRM (Atrial Fibrillation Follow-up Investigation of Rhythm Management)^89^ and RACE (Rate Control versus Electrical Cardioversion for Persistent Atrial Fibrillation)^90^ trials can be explained by withdrawal of oral anticoagulation in patients receiving rhythm control with apparent stable sinus rhythm and by the enhanced pro-arrhythmic effects of antiarrhythmic drug therapy as practiced in the last century.^91^ The AF-CHF (Atrial Fibrillation and Congestive Heart Failure) study compared rhythm control therapy with amiodarone to no rhythm control in patients with AF and a left ventricular ejection fraction < 35% and found no difference in cardiovascular death.^92^ While the neutral effect of amiodarone in AF-CHF is not fully understood, especially in context with the CASTLE-AF (Catheter Ablation versus Standard Conventional Therapy in Patients with Left Ventricular Dysfunction and Atrial Fibrillation) and CASTLE-HTx (Catheter Ablation for Atrial Fibrillation in Patients with End-Stage Heart Failure and Eligibility for Heart Transplantation) trial results,^59,60^ it is possible that the AF burden reduction achieved by amiodarone was not sufficient to reduce cardiovascular events. Furthermore, the primary outcome of AF-CHF, cardiovascular death, was not the most sensitive primary outcome parameter (see prior section), considering the available heart failure treatments applied at the time.^93^ Overall, recent results from randomized trials support the concept that reducing AF burden has a positive effect on outcomes in patients with AF and risk factors.^41^

The outcome-reducing effect of rhythm control is more pronounced in patients with a high comorbidity burden (CHA_2_DS_2_-VASc score ≥ 4),^94^ suggesting an interaction between these two drivers of stroke risk. Providing early rhythm control systematically and subsequently lowering AF burden to patients with a high comorbidity burden^86,94^ results in a more pronounced reduction in cardiovascular outcomes. Similarly, the difference between AF ablation and medical therapy is more pronounced in patients with heart failure and a higher comorbidity burden.^95^ The recently published update of the ACC/AHA/HRS AF guidelines^96^ recognizes AF burden reduction as a therapeutic goal in patients with AF, partially based on these recent findings.

#### Symptoms and quality of life

There is excellent evidence that rhythm control therapy improves AF-related symptoms and quality of life.^5,66,73,97,98^ This effect is more pronounced after AF ablation than on antiarrhythmic drug therapy,^6,73,77,99^ but can be achieved on long-term rhythm control using both modalities.^9^ The outcome-reducing effect of early rhythm control is also found in asymptomatic patients with AF,^10^ suggesting that AF burden reduction may be justified beyond symptom reduction. Clearly, reducing AF-related symptoms, psychological distress,^6^ and other domains of quality of life^5^ remain important treatment goals in all patients with chronic cardiovascular diseases,^100^ including in patients with AF. It is conceivable that symptom reduction follows AF burden reduction, but other mechanisms and factors will interact in this complex treatment domain.

### Interactions between arrhythmia burden and anticoagulation therapy

#### Anticoagulation is not more effective than antiplatelet therapy in patients without atrial fibrillation

Anticoagulation does not prevent strokes in patients without AF, as shown in randomized controlled trials in patients with heart failure,^101,102^ in patients with embolic stroke of undetermined source (ESUS),^103,104^ and in patients with atrial cardiomyopathy^105^ (*Table 2*). Atrial fibrillation detected after an acute stroke (AFDAS) will be detected more often when ECG monitoring is prolonged.^106,107^ Atrial fibrillation detected after an acute stroke can be a first sign of paroxysmal AF but can also occur as a consequence of specific heart–brain interactions triggering transient episodes of AF.^108^ The causal role of short episodes of AFDAS for the stroke event detected with intensified monitoring for longer periods and DDAF including implantable loop recorders, which have increasingly been used for AFDAS search, has remained unclear. Based on detection rates of DDAF in elderly patients with implanted loop recorders,^34,35,48^ patients randomized in the ESUS and heart failure trials included a proportion of patients (20%–30%) with rare and short episodes of DDAF. The clinical AF detection of 3.3% over a median follow-up of 11 months and 7.5% over a median of 19 months after an initial workup to exclude AF on enrolment is consistent with this assumption and with a low AF burden.^109,110^

**Table 2:** Overview of randomized controlled trials in patients with embolic stroke of undetermined source (ESUS) and their relation on outcomes

Study/data set	Age (years)	Women (%)	Comorbidity burden (CHA_2_DS_2_-VASc score)	Event rate of recurrent stroke^a^ (%/patient-year)	Cardiovascular death rate (%/patient-year)
ARCADIA (Kamel *et al.*^105^)					
Anticoagulation (DOAC)	67.8 (10.8)	53.7	4.7 (1.3)	4.4	
Aspirin	68.2 (11.0)	54.9	4.7 (1.3)	4.4	
RE-SPECT ESUS (Diener *et al.*^104^)					
Anticoagulation (DOAC)	64.5 ± 11.4	62.9		4.1	0.4
Aspirin	63.9 ± 11.4	63.4		4.8	0.5
NAVIGATE ESUS (Hart *et al.*^103^)					
Anticoagulation (DOAC)	66.9 ± 9.8	38		5.1 (ischaemic stroke)	1.0
Aspirin	66.9 ± 9.8	39		4.7 (ischaemic stroke)	0.7

Age, women, and CHA_2_DS_2_-VASc score are shown as mean ± standard deviation or median (interquartile range) or number and (percentage) of patients.

DOAC, direct oral anticoagulant; ECG, electrocardiogram; ESUS, embolic stroke of undetermined source.

^a^Stroke definitions vary.

#### Atrial fibrillation burden of device-detected atrial fibrillation, its temporal relation to stroke, and development to electrocardiogram-documented atrial fibrillation

Cardiac implantable electronic devices detect short and rare episodes of DDAF in 10%–30% of patients.^33^ The median AF burden in LOOP was 0.13%^29^ (*Figure 1*). There is no clear temporal relation of DDAF episodes and stroke in ASSERT (Asymptomatic Atrial Fibrillation and Stroke Evaluation in Pacemaker Patients and the Atrial Fibrillation Reduction Atrial Pacing Trial)^111^ or in LOOP,^48^ although a small increase in AF burden can be detected in the month prior to a stroke in patients with DDAF.^112^ Similar to the progression of paroxysmal AF, DDAF advances slowly to ECG-diagnosed AF. In the NOAH-AFNET 6 trial, ECG-detected AF developed in almost 9% per patient-year^12^ and increased to 17% per patient-year in patients with DDAF episodes >24 h at baseline.^13^ Device-detected AF was also strongly associated with development of ECG-documented AF in a meta-analysis with an odds ratio of 5.7 for ECG-documented AF.^113^ The ongoing Find-AF 2 (Intensive Rhythm Monitoring to Decrease Stroke and Systemic Embolism) and AF SPICE (Atrial Fibrillation Screening Post Ischemic Cerebrovascular Events) studies will provide additional randomized data on repeat continuous rhythm monitoring by Holter ECG and standard ECG monitoring post-ischaemic stroke.^114,115^

#### Atrial fibrillation burden and stroke rate in patients with different atrial fibrillation patterns

**Figure 2:**
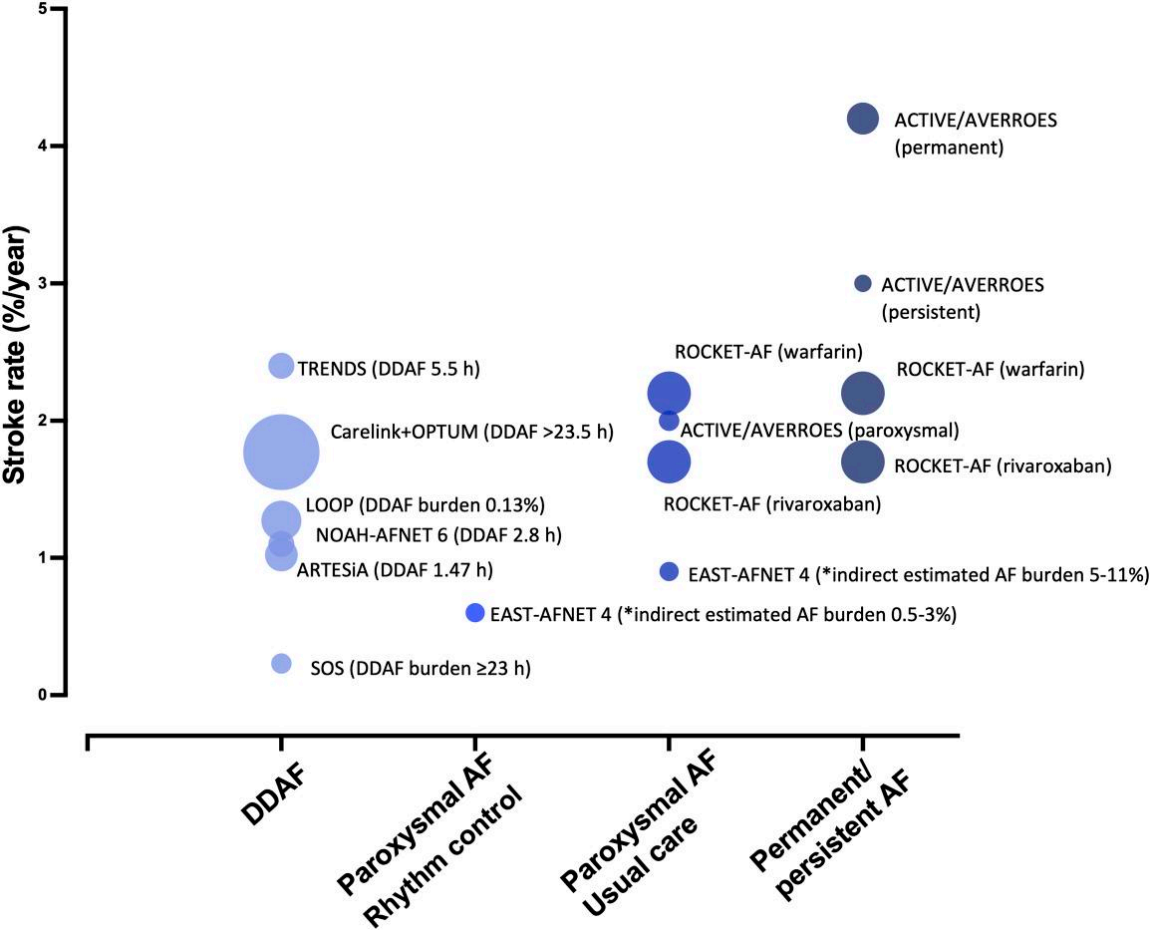
Overview of current randomized controlled trials and studies regarding the correlation of the longest or median device-detected atrial fibrillation and/or atrial fibrillation burden with stroke risk (annual rate). The studies on the right side of of the figure depicts a higher estimated atrial fibrillation burden. In most of the studies, the stroke rate is displayed without anticoagulation. In some studies, the rate of anticoagulation varies as explained below. The figure illustrates concepts and, therefore, is simplified. More details can be found in the cited studies. For the ROCKET-AF study,^116^ the warfarin and rivaroxaban group are shown. In the rivaroxaban group, 17.5% patients had paroxysmal atrial fibrillation and 81.1% persistent atrial fibrillation. In the warfarin group, 17.8% had paroxysmal atrial fibrillation and 80.8% persistent atrial fibrillation. For ARTESiA^14^ and NOAH-AFNET 6,^12^ the median of the longest device-detected atrial fibrillation is displayed. In ARTESiA, patients with device-detected atrial fibrillation > 24 h were excluded. For ARTESiA and NOAH-AFNET 6, ischaemic stroke rate is displayed without anticoagulation. For the Carelink+OPTUM^39^ study, the rate for stroke/systemic embolism is displayed for device-detected atrial fibrillation > 23.5 h in non-anticoagulated patients. For the TRENDS^46^ study, the atrial tachycardia/atrial fibrillation burden is defined as the longest total atrial tachycardia/atrial fibrillation duration on any given day during the prior 30-day window. The rate for stroke/transient ischaemic attack/systemic embolism for the highest device-detected atrial fibrillation duration of 5.5 h is displayed in the plot. In the overall population of the TRENDS study, 20.8% of patients were on warfarin. For the LOOP study,^48^ the atrial fibrillation burden is the cumulative duration of all atrial fibrillation episodes lasting 6 min from the first adjudicated atrial fibrillation episode onwards, divided by total duration of monitoring. The rate for ischaemic stroke/transient ischaemic attack/systemic embolism is displayed in the implantable loop recorder group. In the implantable loop recorder group, in 29.2% participants, oral anticoagulation was initiated. For the SOS study,^44^ the stroke rate for episodes > 23 h duration is displayed. In EAST-AFNET 4,^9^ the rate for stroke for usual care and early rhythm control is displayed. *Indirect estimate; according to Charitos *et al.*,^24^ the approximate atrial fibrillation burden for paroxysmal atrial fibrillation is 5%–11% (usual care) and for persistent/permanent atrial fibrillation 70%–100%. AF, atrial fibrillation; AT, atrial tachycardia; DDAF, device-detected atrial fibrillation; ILR, implantable loop recorder; TIA, transient ischaemic attack

Anticoagulants prevent stroke in patients with ECG-diagnosed AF.^63,67^ The risk of stroke in patients with paroxysmal AF (2%/year without anticoagulation) is lower than the stroke risk in patients with chronic forms of AF (3%/year without anticoagulation; *Figure 2*).^23,43^ The average AF burden in patients with paroxysmal AF is around 5%–11%. Conceptually, the arrhythmia burden should be 100% in patients with persistent and permanent AF. Occasional regression of AF leads to an estimated AF burden of 70%–100% in patients with persistent AF.^24^ Still, the AF burden is ∼10-fold higher in persistent AF than in paroxysmal AF (*Figures 1* and *2*). Rhythm control therapy achieves a 10-fold reduction of AF burden compared with paroxysmal AF without rhythm control (0.5%–3%). Early rhythm control therapy reduces cardiovascular outcomes including stroke in the EAST-AFNET 4 trial to below 1%/year^9^ (*Table 3*). Rate of stroke appears <1%/year after AF ablation, the most effective rhythm control therapy.^118,119^ Whether oral anticoagulation remains effective after successful AF ablation is studied in the ongoing OCEAN (Optimal Anti-Coagulation for Enhanced-Risk Patients Post–Catheter Ablation for Atrial Fibrillation) trial.^120^ This is a rate of stroke that appears similar to the rate of stroke in patients with DDAF receiving anticoagulation (0.7%/year^36^) who have an even lower AF burden than those with AF on rhythm control (0.13%; *Figure 1*).

**Table 3:** Overview of randomized controlled trials regarding rhythm control and their relation of atrial fibrillation burden/duration of the longest device-detected atrial fibrillation episode on outcomes

Study/data set	AF type or pattern (paroxysmal, persistent, permanent) or device-detected AF (DDAF)	Age (years)	Women (%)	Comorbidity burden (CHA_2_DS_2_-VASc score)	AF burden (%/monitored time)	Event rate of stroke (%/patient-year)	Event rate of HF (%/patient-year)	Event rate of CV death (%/patient-year)
Rhythm control studies								
EARLY-AF—3-year follow-up (Andrade *et al.*^76^)								
Antiarrhythmic group	ILR	59.5 ± 10.6^a^	31.2^a^	1 (0, 2)^a^	0.24 (0.01–0.94)^b^	++		
Ablation group		58.2 ± 11.2^a^	28.1^a^	1 (0, 2)^a^	0 (0.00–0.12)^b^	++		
MANTRA-PAF (Nielsen *et al.*^117^)								
Antiarrhythmic group	7-day Holter ECG (3, 6, 12, 18, 24 months)	54 ± 10	28	CHADS_2_ 0–2: 96.6%	9% (at 24 months 90th percentile of AF burden)	++		
Ablation group		56 ± 9	32	CHADS_2_ 0–2: 97.3%	18% (at 24 months 90th percentile of AF burden)	++		
EAST-AFNET 4 (Kirchhof *et al.*^9^)								
Usual care	First episode, paroxysmal, persistent	70.4 ± 8.2	46.5	3.3 ± 1.3		0.9	2.6	1.3
ERC		70.2 ± 8.4	46.2	3.4 ± 1.3		0.6	2.1	1.0

Age and CHA_2_DS_2_-VASC score are shown as mean ± standard deviation or median (interquartile range) or percentage of patients.

++Sample size and observation time were too low in MANTRA-PAF and EARLY-AF to provide meaningful estimates of event rates for outcomes.

AF, atrial fibrillation; CV, cardiovascular; DDAF, device-detected atrial fibrillation, DOAC, direct oral anticoagulant; ECG, electrocardiogram; ERC, early rhythm control; HF, heart failure; ILR, implantable loop recorder; IQR, interquartile range; SE, systemic embolism; SD, standard deviation.

^a^Completed 3-year follow-up.

^b^Median (IQR) percentage of time in AF.

Recent data suggest that circulating biomolecules, including elevated N-terminal pro-B-type natriuretic peptide (NT-proBNP)^121–123^ and elevated bone morphogenetic protein 10,^124–127^ can identify patients at risk of AF and of stroke because these biomolecules are associated with atrial dysfunction and AF.^124,128^ Combinations of these biomolecules can help to predict recurrent AF and may be useful proxies to find patients with a high AF burden. The ongoing STROKESTOP II study is testing whether NT-proBNP can be used to enrich patients undergoing systematic AF screening.^129^

### From binary atrial fibrillation detection to quantification of atrial fibrillation burden

The current ‘yes/no’ binary definition of AF is established in clinical practice and easily implemented in different healthcare settings, including initiation of anticoagulants in non-specialist settings.^130^ The data discussed here illustrate that a more granular view can refine risk prediction, considering AF burden as a contributor to stroke, heart failure, and other outcomes in AF. Despite the remaining uncertainty about its estimation in patients, integrating AF burden into treatment decisions in patients with AF has the potential for more precise estimation of risk and better selection of therapies (*Graphical Abstract*).

#### Clinical implications

The association of AF burden with cardiovascular events needs more research. Based on the data available so far, the following clinical implications can be inferred:

1. AF burden affects cardiovascular events in patients with AF. Therefore, most treatment decisions in patients with ECG-documented clinical AF remain valid.
2. Reducing AF burden, typically using rhythm control therapy, should be a therapeutic goal in patients with AF at risk of stroke and heart failure, as long as it can be achieved safely. A first step towards this is reflected in the recent update of the ACC/AHA/HRS AF guidelines.^96^
3. Patients with a very low AF burden appear to have a risk of stroke and heart failure that is relatively low and not dissimilar to patients without AF. Treatment decisions should be individualized, considering the effectiveness and safety of anticoagulation and rhythm control therapy.
4. Atrial fibrillation burden can be quantified in patients with implanted devices. A uniform, cross-manufacturer standard to measure time in AF, and to calculate AF burden, would greatly help clinicians caring for patients with implanted devices.
5. The AF burden in patients with AF detected by consumer electronics is typically low unless they have ECG-diagnosed AF. Monitoring for ECG-documented AF appears reasonable in these patients at present.
6. When only intermittent rhythm monitoring is available, AF burden can be estimated by the time in AF divided by the monitoring time, or by days in AF divided by monitored days. These estimates need to be applied with care, considering their uncertainty.
7. When longer monitoring durations are available and AF burden cannot be exactly calculated, the duration of the longest episode can be used as a proxy for AF burden.

#### Knowledge gaps and research opportunities

To better understand the relation between AF burden and outcomes, quantification of AF burden is needed in more patients, and its relation to outcomes needs to be analysed (*Table 4*). Fortunately, the development of innovative data science tools^131^ and the development of simple devices capable of continuous rhythm monitoring^15,18^ now enable such research in broader patient populations. Conventionally agreed stroke risk thresholds, e.g. a CHA_2_DS_2_-VASc score ≥ 3^36^ or >4^40^ or detection of episodes of DDAF ≥ 24 h duration,^13^ may not be sufficient to identify patients with DDAF at high risk of stroke. Linkage of continuous monitoring for AF burden with outcome data can define AF patterns (number of episodes, episode duration) and AF burden thresholds that constitute a risk of stroke in interaction between comorbidity burden. Such research should integrate additional risk modifiers including genetic information,^132^ circulating biomolecules,^127,132,133^ and imaging data. Such research will also be able to delineate the amount of AF burden reduction that affects outcomes and to identify AF burden thresholds, most likely in interaction with clinical and other factors increasing risk of AF-related complications, that identify patients with a clear benefit of anticoagulation therapy. Quantitative proxies for arrhythmia burden and for comorbidity severities should be defined to identify tipping points that justify a change in therapy. These will be different for stroke, heart failure, or dementia.

**Table 4:** Gaps in knowledge and challenges

Gaps of knowledge/challenges	Potential solutions/studies needed
Definition and reliable quantification of AF burden	Uniform definition of AF burden with clinical meaning and easy-to-implement quantification in practice and research
	Creation of innovative data science tools for AF burden assessment
	Development of simple devices capable of continuous rhythm monitoring
	Validating technology that quantifies AF burden
	Standardizing AF burden detection algorithms (cross-manufacturer standard measurement) and quantification in different devices and consumer electronics
AF burden and outcomes: better understanding of association of AF burden and outcomes	Individualization of treatment decisions, considering the effectiveness and safety of anticoagulation and rhythm control therapies
	Generation of data on AF burden in relation to stroke/TIA, heart failure, dementia, mortality, quality of life, and other outcomes
	Systematic implementation of AF burden as (intermediate) outcome
	Temporal relation of AF burden with adverse events
	Association of AF burden with outcomes in distinct settings, e.g. post-ablation, consumer-based monitoring, and post-stroke
Effect of ablation reducing AF burden on outcomes	Secondary and meta-analysis of existing studies Ongoing and planned trials, e.g. CABA-HFPEF (NCT05508256) and EASThigh-AFNET 11 (NCT06324188)
AF burden thresholds requiring therapy (e.g. anticoagulation)	Need of specific studies/secondary analyses of RCTs
	Identifying patients/subgroups at higher risk
	Integrating genetic information, circulation biomarker, and imaging modalities
	Reduced anticoagulation justified in low arrhythmia burden, e.g. ongoing studies, OCEAN, REACT-AF

#### Ongoing trials

Whether a low arrhythmia burden is a justification to reduce therapy is tested in ongoing studies. The OCEAN trial will test whether anticoagulation can be withheld after successful AF ablation in patients with and stroke risk factors.^120^ Based on the hypothesis that only longer episodes of AF increase thromboembolic risk in paroxysmal AF with limited comorbidities and arrhythmia substrate, the REACT-AF (Rhythm Evaluation for Anticoagulation Therapy for Atrial Fibrillation) trial will compare continuous anticoagulation with a smartwatch-guided time-delimited anticoagulation regime that is confined to periods with a high AF burden.^134^ At least two trials, ongoing or planned, will evaluate the effect of AF burden reduction by AF ablation on outcomes in patients with AF and HFpEF (CABA-HFPEF, NCT05508256) and in patients with AF and multiple comorbidities (EASThigh-AFNET 11, NCT06324188). For AF screening, enrichment strategies using circulating biomolecules associated with AF, stroke, and heart failure (BMP10, NT-proBNP, troponin, angiopoietin-2, fibroblast growth factor-23, and others) may be useful. Other ongoing AF screening studies such as the SAFER (Screening for Atrial Fibrillation with ECG to Reduce Stroke, ISRCTN72104369) study with intermittent handheld device tracings over 3 weeks in over 100 000 individuals^135^ or the AMALFI (Active Monitoring for Atrial Fibrillation, ISRCTN15544176) study with 2-week patch device monitoring will provide further data on threshold for treatment in screen-detected AF.

### Limitations

The review draws data from different research areas and combines them to propose the concept of AF burden and AF burden reduction. We did not see a mechanism to formalize the data analysis for this purpose, e.g. as meta-analysis. Quantitative details are reported where available. Patients with AF experience reduced quality of life due to a complex interplay of symptoms, anxiety, fear of AF-related adverse outcomes, and concerns about recurrence. Rhythm control therapy is clearly indicated to mitigate these symptoms.^1,96^ Many of the details required for interpretation of AF burden in clinical practice await evaluation and quantification. We hope that this review triggers more research and technological innovation to better measure and understand AF burden and its role in the diagnosis and management of patients with AF.

### Summary

The enhanced ability to quantify the burden of AF by implanted devices and wearables demonstrates a large spread of DDAF episodes for duration, frequency, dispersion, and development. A burden-based description of temporal AF patterns related to outcomes and treatment strategies can improve risk prediction based on earlier classification into paroxysmal, persistent, and long-standing persistent AF. Early rhythm control can reduce AF burden and is associated with improved outcomes. Similar effects can be seen for AF ablation in patients with heart failure. These interventional data provide first evidence that AF burden reduction can improve outcomes. Current data indicate that short, infrequent episodes of DDAF carry a low stroke risk and may not require anticoagulation therapy. Individual decisions on anticoagulation initiation need to be balanced considering the individual risk of stroke and bleeding and will likely include AF burden in the near future. Device-detected AF episodes can develop into ECG-diagnosed AF over time. This development is slow, comparable with the long time from paroxysmal to persistent AF. Regular ECGs are useful for clinical practice because the transition from DDAF to ECG-documented AF significantly increases the evidence base available for guideline-directed AF therapy.

## Supplementary data

Supplementary data are not available at *European Heart Journal* online.

## Data Availability

No data were generated or analysed for or in support of this paper.
